# Association between Beta Oscillations from Subthalamic Nucleus and Quantitative Susceptibility Mapping in Deep Gray Matter Structures in Parkinson’s Disease

**DOI:** 10.3390/brainsci13010081

**Published:** 2023-01-01

**Authors:** Mangui Lin, Guoen Cai, YongJie Li, Yifang Sun, Yang Song, Guofa Cai, Rifeng Jiang

**Affiliations:** 1 College of Information Engineering, Guangdong University of Technology, Guangzhou 510006, China; 2 Department of Neurology, Fujian Medical University Union Hospital, Fuzhou 350001, China; 3 Department of Radiology, Fujian Medical University Union Hospital, Fuzhou 350001, China; 4 MR Scientific Marketing, Siemens Healthcare, Shanghai 200126, China

**Keywords:** Parkinson’s disease, brain iron deposition, beta oscillations, local field potentials

## Abstract

This study aimed to investigate the association between beta oscillations and brain iron deposition. Beta oscillations were filtered from the microelectrode recordings of local field potentials (LFP) in the subthalamic nucleus (STN), and the ratio of the power spectral density of beta oscillations (PSDXb) to that of the LFP signals was calculated. Iron deposition in the deep gray matter (DGM) structures was indirectly assessed using quantitative susceptibility mapping (QSM). The Unified Parkinson’s Disease Rating Scale (UPDRS), part III, was used to assess the severity of symptoms. Spearman correlation coefficients were applied to assess the associations of PSDXb with QSM values in the DGM structures and the severity of symptoms. PSDXb showed a significant positive correlation with the average QSM values in DGM structures, including caudate and substantia nigra (SN) (*p* = 0.008 and 0.044). Similarly, the PSDXb showed significant negative correlations with the severity of symptoms, including axial symptoms and the gait in the medicine-off state (*p* = 0.006 for both). The abnormal iron metabolism in the SN and striatum pathways may be one of the underlying mechanisms for the occurrence of abnormal beta oscillations in the STN, and beta oscillations may serve as important pathophysiological biomarkers of PD.

## 1. Introduction

As one of the most common neurodegenerative diseases, Parkinson’s disease (PD) commonly exhibits both motor and non-motor symptoms; resting tremors, bradykinesia, rigidity, and postural instability are common motor symptoms [1,2,3]. The growing incidence of PD has brought a considerable burden to society and significant distress to both patients and caregivers.

For advanced PD patients, deep brain stimulation (DBS) of the subthalamic nucleus (STN) is an effective way of relieving their motor symptoms, [4] which also provides a precious opportunity to record, in vivo, the related neuronal activity in high spatial and temporal resolution through microelectrode recordings (MERs) [2]. During neuronal activity, beta oscillations have been confirmed to be excessively increased in PD patients [5,6] and have a strong correlation with the severity of bradykinesia [6,7,8]. Furthermore, while on levodopa and DBS, beta oscillation activity decreases while clinical symptoms improve [9,10]. Thus, beta oscillations are supposed to inhibit normal movement and have a close relationship with bradykinesia and rigidity in PD [11,12].

On the other hand, iron homeostasis maintains normal physiological brain function, whereas the misregulation of iron homeostasis will lead to neurotoxicity through different mechanisms [13]. Excess iron has the ability to form reactive oxygen species, which damage DNA and proteins irreversibly, which eventually leads to iron-mediated cell death [13,14,15,16,17]. Furthermore, increased iron is seen in the substantia nigra (SN) at postmortem in PD patients, indicating excess iron in the brain also plays an important role in the crucial pathophysiological pathways that are specific to PD [14,18]. Therefore, iron deposition contributes to the mechanism of brain damage in PD patients.

In PD studies, the evaluation of in vivo brain iron has become a hotspot. Magnetic resonance imaging (MRI) can detect iron due to its high magnetic susceptibility. Recently, the quantitative susceptibility mapping (QSM) technique has provided quantitative estimates of iron distribution in the brain at a voxel level [19]. In deep gray matter (DGM) structures, QSM has reliably shown the ability to quantify changes in iron content [20,21,22]. Several postmortem studies have demonstrated significant correlations between QSM contrast and the histochemical measurement of iron [20,23,24,25,26,27,28]. Thus, QSM is a noninvasive method capable of reliably measuring changes in iron content in DGM structures.

Taken together, we hypothesize that there may exist an association between beta oscillations from STN and brain iron deposition in DGM structures in PD. However, there is a lack of studies discussing their relationship. In this study, we aimed to reveal the potential association between beta oscillations and brain iron deposition, which may shed more light on the underlying pathogenesis of PD.

## 2. Materials and Methods

### 2.1. Participants

**Figure 1:**
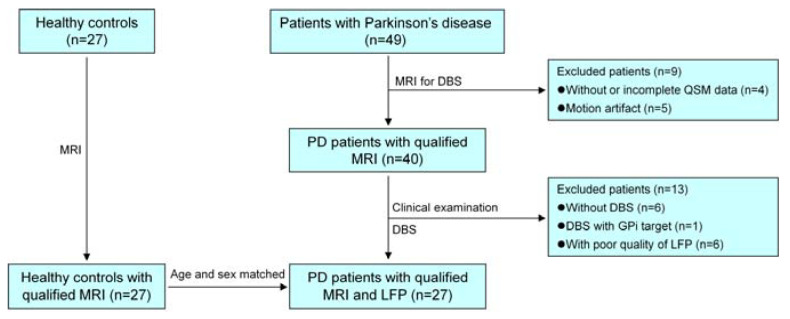
The screening process for including PD patients and healthy controls. MRI = magnetic resonance imaging, PD = Parkinson’s disease, DBS = deep brain stimulation, QSM = quantitative susceptibility mapping, GPi = globus pallidus interna, LFP = local field potential.

From February 2019 to November 2021, 49 primary PD patients were prospectively enrolled in the research. Of the 49 patients, 9 were excluded due to lacking complete QSM data (*n* = 4) or obvious artifacts of head motion (*n* = 5), and another 13 were further excluded for the following reasons: (1) 6 without DBS; (2) 1 with DBS of the globus pallidus interna (GPi) target (not the STN target); and (3) 6 with poor quality of local field potentials (LFP). Finally, 27 patients with qualified STN-LFP and QSM (16 males and 11 females; age: 59.48 ± 8.32 years) and 27 age- and gender-matched healthy controls (HC) (12 males and 15 females; age: 59.93 ± 7.13 years) were included in this study. The screening process for the included PD patients and HCs is shown in Figure 1. This study was approved by the Institution Review Board of Fujian Medical University Union Hospital, and written informed consent was obtained from all the participants.

### 2.2. Clinical Information

Clinical characteristics, including age, gender, disease duration, and levodopa equivalent daily dose (LEDD), were recorded. Motor impairment was assessed using the Hoehn and Yahr (H-Y) stages and the Unified Parkinson’s Disease Rating Scale (UPDRS), part III, in both the medicine-on (med-on) and medicine-off (med-off) states. The subitems of UPDRS part III include tremor (items 20–21), rigidity (item 22), bradykinesia (items 23–26), axial symptoms (items 27–30), and gait (item 29) [29,30].

### 2.3. MERs Data and LFP Analysis

Of the 27 microelectrode recordings from DBS, 18 were recorded with the NeuroNav Physiological Navigation system 4.5.3 (Alpha Omega Engineering Ltd., Nazareth, Israel) and tungsten electrodes (STR-007080-10, Alpha Omega Engineering Ltd., Israel), while the other 9 were recorded with the StealthStation Neuro-Navigational platform (Medtronic Ltd., Minneapolis, MN, USA) and tungsten electrodes (STR-007080-10, Alpha Omega Engineering Ltd., Nazareth, Israel). The electrophysiological signals of STN were band-pass filtered [31]. The LFP recording began when the electrode reached 10 mm above the specified target, and the electrode advanced with 1 mm steps [31]. After the electrode tip entered the dorsal edge of the STN, the step size of the advancing electrode was reduced to 0.5 mm [31]. When the tip of the electrode entered the substantia nigra, the recording was ended.

**Figure 2:**
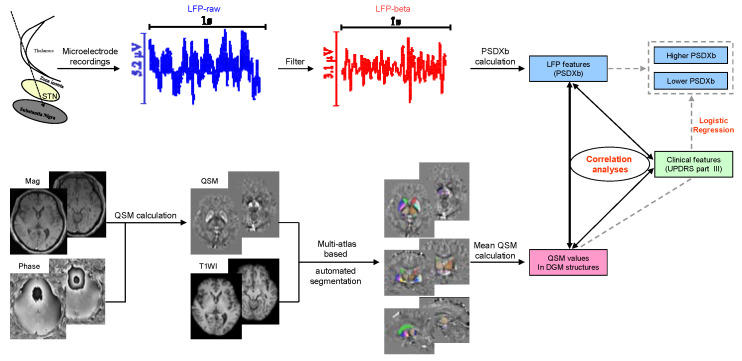
Flowchart for correlation analysis and logistic regression analysis of the LFP features, QSM features, and clinical features. STN = subthalamic nucleus, LFP = local field potential, PSDXb = the ratio of the power spectral density of beta oscillations to that of the LFP signals, UPDRS = Unified Parkinson’s Disease Rating Scale, Mag = magnitude, QSM = quantitative susceptibility mapping, T1W1= T1 weighted image, DGM = deep gray matter.

The LFP signals selected were processed offline using MATLAB (version 2016b, MathWorks Corporation, Natick, MA, USA) and EEGLAB (v2020.0, https://sccn.ucsd.edu/eeglab (accessed on 6 June 2022)). As the activity of LFP signals is mainly concentrated in the low-frequency band, the LFP signals were initially band-pass filtered (0.1–200 Hz) and subsequently notch filtered to eliminate interference (50 Hz) and its harmonics (at 100,150,200 Hz). Then, the LFP signals were down-sampled to 500 Hz and reviewed to discard artifacts. To normalize the LFP signals, a Z-score was used. After normalization, all the signals were further filtered to obtain the beta (13–30Hz) oscillations of LFP, as shown in Figure 2. The power spectral density (PSD) of the LFP signals and filtered beta oscillations were calculated with Welch approaches, which used a function in MATLAB called pwelch. Specifically, to reduce edge effects, a fast Fourier transform with a 1 s Hanning window and a 0.5 s overlap was used to calculate the PSD. Then, the PSDXb, defined as the ratio of the PSD of beta oscillations to that of LFP signals, was calculated. The PSDXbs of the left and right sides were averaged for further analysis. In addition, the average PSDXb was used as a cut-off value to divide the PD patients into patients with a lower PSDXb (*n* = 13) and those with a higher PSDXb (*n* = 14).

### 2.4. MRI Data Acquisition and Processing

All the subjects underwent both structural MRI and QSM imaging on a 3T MR scanner (MAGNETOM Prisma, Siemens Healthcare, Erlangen, Germany) with a 64-channel head coil. The structural MR imaging protocols included sagital T1-weighted magnetization-prepared rapid gradient-echo sequence (T1-MPRAGE) with a voxel size of 0.9375 × 0.9375 × 0.9 mm^3^ and axial and coronal T2-weighted (T2W) fast spin-echo (FSE) images. QSM imaging was based on 3D flow-compensated multi-echo gradient-echo (GRE) images in the axial plane (TR = 35 ms; first TE = 6.67 ms; uniform echo spacing = 6.24 ms; last TE = 25.39 ms; number of echoes = 4; FA = 15°; FOV = 280 × 320 mm^2^; voxel size = 0.72 × 0.72 × 2 mm^3^). 

STIsuite (https://people.eecs.berkeley.edu/~chunlei.liu/software.html (accessed on 6 June 2022)) was used to calculate the QSM map. T1-MPRAGE images were co-registered to the QSM images by rigid-body-registering the first echo magnitude image from the GRE pulse sequence using the SPM12 (www.fil.ion.ucl.ac.uk/spm/software/spm12 (accessed on 6 June 2022)) [32] and skull-stripped by multiplying with a brain binary mask made in mricron (https://www.nitrc.org/projects/mricron/ (accessed on 6 June 2022)) based on the first echo magnitude image from the GRE pulse sequence. The co-registered, skull-stripped QSM and T1WI entered an automated multi-atlas segmentation pipeline using both QSM and T1 contrast to delineate the DGM structures [33]. Subsequently, the DGM structures were automatically segmented, as shown in Figure 2, and average QSM values of the following regions were extracted: bilateral caudate, internal globus pallidus (GPi), external globus pallidus (GPe), putamen, subthalamic nucleus (STN), substantia nigra (SN), red nucleus (RN), and dentate nucleus (DN). The average QSM values of the left and right sides were averaged for further analysis.

### 2.5. Statistics Analysis

The statistical analysis was performed using SPSS (version 26.0, IBM Corporation., Armonk, NY, USA). The clinical features, PSDXb, and QSM values of the PD patients and QSM values of the HCs were described as median and interquartile ranges. Pearson’s chi-squared test (X^2^) was conducted to compare the differences in gender, and the Mann–Whitney U Test was used to compare the differences in age between PD patients and HC participants. To assess the normality assumptions of feature distribution, the Kolmogorov–Smirnov test was used, and normality assumptions were not held for part of the variables. Therefore, the Mann–Whitney U Test was used to compare the differences in QSM values between the PD patients and HC participants. Spearman correlation analysis was applied to assess the correlation between every two LFP features, QSM values, and clinical features, and false discovery rate (FDR) correction using the Benjamini and Hochberg method was applied to correct the p-values for the multiple tests. Binary logistic regression analysis was used to combine features to create a regression equation and calculate the corresponding prediction probability for the PSDXb level. Receiver operating characteristic (ROC) curves were constructed to assess the corresponding diagnostic performance of each effective feature alone and the prediction probability for separating different PSDXb levels. Significance threshold was set to 0.05 in the statistical analysis.

## 3. Results

### 3.1. Characteristics of Participants

The demographic and clinical information of each of the 27 PD patients is shown in Table 1. In addition, 27 age- and gender-matched participants were also recruited as HCs. The descriptive statistics of the demographic and clinical information, as well as the comparisons between the two groups, are presented in Table S1, and no significant difference was found in age and gender between the PD patients and HCs.

### 3.2. QSM Comparison between HCs and PD patients

Compared with those of the HCs, the bilateral average QSM values in the SN and RN were significantly higher in PD patients (*p* = 0.048 and 0.018, respectively). In contrast, no significant difference was found for the bilateral average QSM values in the other DGM structures (*p* > 0.05 for all), as shown in Table S2.

### 3.3. Correlation between PSDXb and QSM Values

The results of the correlation analysis between the bilateral average PSDXb and bilateral average QSM values are presented in Table 2. Significant positive correlations with the bilateral average PSDXb were found for the bilateral average QSM values in the caudate (rho = 0.582, *p* = 0.001) and SN (rho = 0.480, *p* = 0.011), and the FDR-corrected p-values were 0.008 and 0.044, respectively. In contrast, no significant correlation was found between the bilateral average PSDXb and the bilateral average QSM values in the other DGM structures (*p* > 0.05 for all).

The results of the correlation of the PSDXbs on the left and right sides with QSM values on the corresponding sides are presented in Table S3. On the left side, a significant positive correlation with the PSDXb was found for the QSM values in the caudate (rho = 0.555, *p* = 0.003), with an FDR-corrected *p*-value of 0.024. Similarly, on the right side, a significant positive correlation with the PSDXb was also found for the QSM values in the caudate (rho = 0.546, *p* = 0.003), with an FDR-corrected *p*-value of 0.024. In contrast, no significant correlation was found between the PSDXb and the QSM values in the other DGM structures (*p* > 0.05 for all).

### 3.4. Correlation between PSDXb and UPDRS Part III

The results of the correlation analysis between the bilateral average PSDXb and UPDRS part III are shown in Table 3. For the condition of medicine-off, significant negative correlations with the bilateral average PSDXb were identified for the axial symptoms (rho = −0.603, *p* = 0.001) and gait (rho = −0.599, *p* = 0.001), with an FDR-corrected *p*-value of 0.006 for both. In contrast, no significant correlation with the bilateral average PSDXb was found for UPDRS part III and its other subitems. such as tremor, rigidity, or bradykinesia (*p* > 0.05 for all). For the condition of medicine-on, no significant correlation with the bilateral average PSDXb was found for UPDRS part III or its subitems (*p* > 0.05 for all).

### 3.5. Correlation between UPDRS Part III and Bilateral Average QSM Values

The results of the correlation analysis between UPDRS part III and the bilateral average QSM values are listed in Table S4. For both the medicine-off and medicine-on conditions, no significant correlation was found between UPDRS part III and the bilateral average QSM values in all the DGM structures (*p* > 0.05 for all).

### 3.6. Logistic Regression and Diagnostic Performance Evaluation

The bilateral average PSDXb of the PD patients, 0.107, was used as a cut-off value to divide the PD patients into patients with a lower PSDXb (*n* = 13) and those with a higher PSDXb (*n* = 14). The most significant features—axial symptoms (med-off) and bilateral average QSM values in the caudate—were selected and combined by creating a binary logistic regression equation for the PSDXb level, as shown in Equation (1), and the corresponding prediction probability was subsequently calculated. It was found that the bilateral average QSM value in the caudate was a significant predictor of the PSDXb level (*p* = 0.044), and both the odds ratio (OR) and 95% confidence interval (CI) were 18.233 (1.079–308.185) for each 0.01 increase in the QSM value. In contrast, axial symptoms (med-off) were not a significant predictor (*p* = 0.312), and the OR (95%CI) was 0.800 (0.519–1.232).

**Figure 3:**
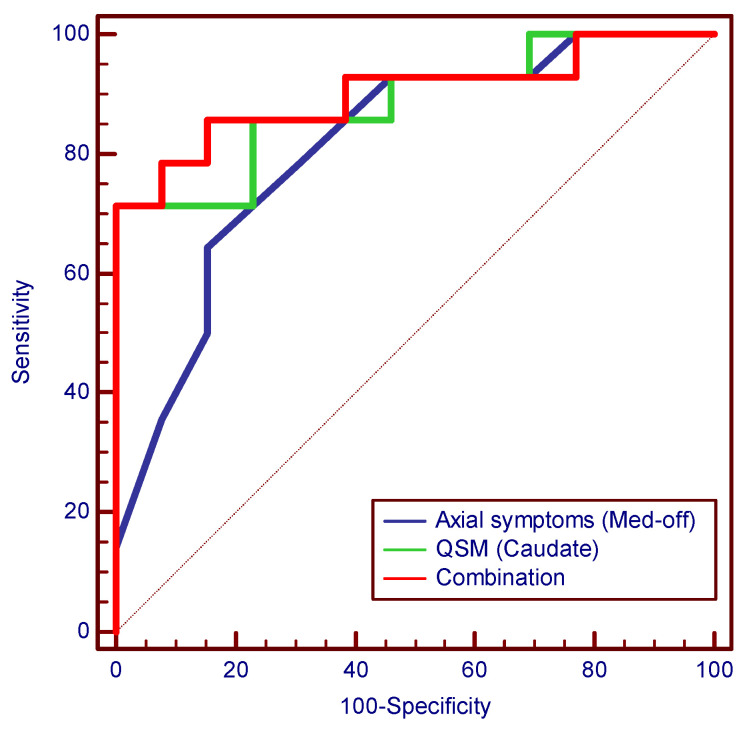
ROC curves for separating the different PSDXb levels of significant features. The combination in the figure indicates the prediction probability of a binary logistic regression model combining axial symptoms (med-off) and the average QSM value in the caudate for the PSDXb levels. PSDXb = the ratio of the power spectral density of beta oscillations to that of the LFP signals, QSM = quantitative susceptibility mapping.

The results of the ROC analyses of the significant features used to separate different PSDXb levels are presented in Table 4, and the representative ROC curves are shown in Figure 3. The significant clinical features and bilateral average QSM values differentiated different PSDXb levels with AUCs of 0.709–0.885. The QSM in the caudate has a higher performance among QSM values, with an AUC of 0.885, a sensitivity of 0.714, and a specificity of 1.000. Similarly, axial symptoms (med-off) have a higher performance among clinical features, with an AUC of 0.813, a sensitivity of 0.643, and a specificity of 0.846. The combination of the above two features—the prediction probability of the logistic regression equation—showed the highest AUC of 0.901, with a sensitivity of 0.714 and a specificity of 1.000.

ln(p/(1 − p)) = 290.324 × QSM (caudate) − 0.223 × axial symptoms (med-off) − 8.080(1)

Logistic regression equation of axial symptoms (med-off) and QSM (caudate) for the prediction of the PSDXb level. In the equation, p indicates the probability that a higher PSDXb is a case, while (1-p) indicates the probability that it is a non-case.

## 4. Discussion

Few studies link beta oscillations in STN to iron deposition in DGM structures in patients with PD. To the best of our knowledge, the present article is the first human experimental evidence of a direct relationship between beta oscillations and iron deposition. We found that beta oscillations in STN are significantly correlated with the QSM value in DGM structures and the severity of symptoms. We highlight that PSDXb of the beta oscillations showed significant correlations with the QSM value in DGM structures, especially the caudate and SN. Furthermore, higher levels of QSM in the caudate were found to be associated with a greater PSDXb independently of the severity of symptoms in PD patients.

There is a growing body of evidence in recent decades suggesting that iron depositions in DGM structures play an important role in the pathology of PD [21,22]. In vitro studies report that excessive iron accumulation in cultured dopaminergic cells can trigger ferroptosis [34]. For example, ferroptosis was induced in cultured SH-SY5Y cells exposed to neurotoxins such as 1-methyl-4-phenylpyridinium (MPTP) and 6-hydroxydopamine, whereas adding ferrostatin-1, an inhibitor of ferroptosis, to these cultured cells markedly reduced ferroptosis [35,36]. Similar findings were also observed in 1-methyl-4-phenyl-1,2,3,6-tetrahydropyridine-induced PD mouse models [37] and α-SynA53T PD mice [34]. Furthermore, some evidence demonstrates a potential role for iron accumulation in α-Syn aggregation [38,39]. For example, high levels of iron upregulate the levels of α-Syn [40] and produce free radicals, leading to cell damage [41]. In contrast, iron displacement therapy can effectively improve the motor symptoms of PD patients in clinical studies [42]. For example, deferiprone has been reported to have beneficial effects on PD patients in clinical studies [43]. On the other hand, LFP recorded from the striata of parkin-mutant mice robustly displayed amplified beta oscillations [44], and the increased striatal beta oscillations in MPTP mice are comparable to those of PD patients [45]. Taken together, we consider that the STN beta oscillations are associated with iron deposition in PD. The possible mechanism is that increased iron depositions will lead to the death of dopamine neurons in the substantia nigra and striatum pathways, and reduced dopamine release means a relative increase in acetylcholine in the substantia nigra and striatum, which leads to the increased release of gamma-aminobutyric acid (GABA). As an inhibitory neurotransmitter, GABA enhances inhibition to GPe, resulting in a decrease in the inhibitory function of GPe to STN, finally leading to the hyperfunction of STN and an increase in pathological β oscillation.

In this study, QSM values were found to be higher in the SN and RN in PD patients compared with those in HCs. The QSM values were high in RN and SN, suggesting iron deposition in these two regions of PD patients. The results of higher QSM values in SN were consistent with previous studies [22,46,47,48,49]. The higher QSM values in RN were consistent with some of the previous studies, [46,47] which remains controversial. Compared with HCs, in PD patients, significant differences in the QSM values in DGM structures may or may not occur except in the SN [22,46,47,48,49]. In our study, for PD patients, iron depositions in DGM structures, except the SN and RN, were not significantly different compared with those of the HCs; the possible reason was the small sample size, which caused no significant difference to be found in the statistics. Increased iron deposition will cause neurotoxicity and even iron-mediated cell death through different mechanisms [48]. The degeneration and death of dopaminergic neurons subsequently reduce dopamine secretion in the SN, thus resulting in symptoms such as resting tremors, bradykinesia, rigidity, and postural instability in PD patients.

Interestingly, the correlation between the PSDXb with the QSM value in the caudate was stronger than that of the other DGM structures in this study. The possible explanation is that the caudate, as part of the striatum, may have more direct regulation, as shown in Figure S1 in the Supplementary Materials. In addition, previous studies reported a significant association between caudate abnormality with dyskinesias and executive dysfunctions [50,51,52]; therefore, the QSM value in the caudate may also be associated with dyskinesias and executive dysfunctions. However, a caudate-related clinical assessment was not performed in this study. Therefore, the association the QSM values in the caudate have with dyskinesias and executive dysfunctions will be refined in our future studies.

In this study, STN beta oscillations were also found to be significantly associated with the severity of motor symptoms [53,54], especially for the axial symptoms and gait (med-off), supporting the idea that beta oscillations may serve as important pathophysiological biomarkers of PD [55,56]. In line with other studies [53], this study found beta oscillations in the STN were positively associated with axial symptoms in PD. In addition, a previous study found that beta-oscillating unit activity was also associated with rigidity [53]. However, gait, but not rigidity, was found to be positively associated with beta oscillations in this study, which may be a complementary finding.

In addition, we further found that iron deposition in the caudate has a significant effect on the PSDXb level of beta oscillations. The QSM value in the caudate was a significant predictor of the PSDXb level independently of axial symptoms (med-off). The QSM in the caudate also showed better performance in the classification of PSDXb levels with an AUC of 0.885. A logistic regression model combining multiple factors further improved the performance, and the AUC increased to 0.901.

There are some limitations in our study. First, the sample size of this study was relatively small, and the included patients were quite heterogeneous in terms of disease duration. Second, the LFP activity was invasively recorded in PD patients; however, it was not available from the HCs. Therefore, the difference in LFP features was not compared between these two groups. Third, the LFP activity was recorded using two different instruments. However, normalization was used to eliminate the differences prior to the statistical analysis. Finally, the iron content of DGM structures cannot be measured directly; therefore, it is still a limitation that iron deposition was indirectly evaluated using QSM. However, as far as we know, QSM is currently the best noninvasive method for evaluating iron deposition in the DGM structures in PD patients.

In conclusion, the PSDXb of beta oscillations from the STN has strong associations with QSM values in DGM structures, especially in the caudate and SN, indicating that abnormal iron metabolism in the SN and striatum pathways may be one of the underlying mechanisms of the occurrence of abnormal beta oscillations in the STN. Moreover, the PSDXb of beta oscillations shows strong associations with the severity of symptoms, supporting the idea that beta oscillations may serve as important pathophysiological biomarkers of PD.

## Figures and Tables

**Table 1:** Demographic and clinical characterizations for the PD patients.

Patient ID	Gender	Age (Years)	Disease Duration (Years)	LEDD (mg)	H-Y	UPDRS Part III	
						Med-Off	Med-On
1	F	60	10	900	3	59	23
2	F	71	15	1075	4	70	48
3	F	64	8	799	3	75	35
4	M	64	5	844	2.5	44	30
5	F	59	10	225	5	53	9
6	M	67	12	800	3	46	29
7	M	54	5	574	2.5	42	16
8	F	63	6	450	2	46	28
9	M	63	12	375	3	47	22
10	M	60	11	650	3	57	41
11	F	49	5	1450	2.5	56	13
12	M	43	7	604	3	60	28
13	M	74	4	474	3	38	25
14	F	62	10	798	4	60	38
15	F	60	8	732	3	39	25
16	M	60	10	250	3	33	10
17	M	55	9	1750	3	50	28
18	M	65	20	499	3	53	22
19	F	54	7	1148	3	55	26
20	M	67	10	997	2.5	41	25
21	M	56	13	625	4	79	34
22	F	63	16	1200	2.5	63	14
23	M	59	7	887	3	49	17
24	F	69	17	675	3	50	24
25	M	58	9	700	3	62	26
26	M	54	8	1125	3	69	30
27	M	33	6	1450	3	37	8

LEDD = levodopa equivalent daily dose, H-Y = Hoehn and Yahr stages, UPDRS = Unified Parkinson’s Disease Rating Scale.

**Table 2:** Results of the correlation analysis between the bilateral average PSDXb and bilateral average QSM values in DGM structures.

Bilateral Average QSM Value	Bilateral Average PSDXb		
	rho	*p*	*p* (FDR-Corrected)
Caudate	0.582	0.001 *	0.008 *
GPi	0.332	0.091	0.146
GPe	0.346	0.077	0.146
Putamen	0.203	0.309	0.412
STN	0.363	0.063	0.146
SN	0.480	0.011 *	0.044 *
RN	−0.004	0.983	0.983
DN	−0.105	0.602	0.688

* represents a statistical correlation (*p* < 0.05). DGM = deep gray matter, PSDXb = the ratio of the power spectral density of beta oscillations to that of the LFP signals, QSM = quantitative susceptibility mapping, FDR = false discovery rate, GPi = internal globus pallidus, GPe = external globus pallidus, STN = subthalamic nucleus, SN = substantia nigra, RN = red nucleus, DN = dentate nucleus.

**Table 3:** Results of the correlation analysis between the bilateral average PSDXb and UPDRS part III.

UPDRS Part III	Bilateral Average PSDXb		
	rho	*p*	*p* (FDR-Corrected)
UPDRS part III (med−off)	−0.277	0.162	0.648
Tremor	−0.078	0.698	0.877
Rigidity	−0.050	0.804	0.877
Bradykinesia	−0.088	0.664	0.877
Axial symptoms	−0.603	0.001 *	0.006 *
Gait	−0.599	0.001 *	0.006 *
UPDRS part III (med−on)	0.073	0.718	0.877
Tremor	−0.063	0.754	0.877
Rigidity	0.161	0.424	0.877
Bradykinesia	0.019	0.926	0.926
Axial symptoms	0.051	0.801	0.877
Gait	−0.054	0.788	0.877

* represents a statistical correlation (*p* < 0.05). PSDXb = the ratio of the power spectral density of beta oscillations to that of the LFP signals, FDR = false discovery rate, UPDRS = Unified Parkinson’s Disease Rating Scale.

**Table 4:** Results of ROC analyses of the significant features used to separate different PSDXb levels.

Metric	Lower PSDXb (*n* = 13) vs. Higher PSDXb (*n* = 14)					
	AUC (95% CI)	Cut-Off	Sensitivity (95% CI)	Specificity (95% CI)	PPV (95% CI)	NPV (95% CI)
Axial symptoms (med-off)	0.813 (0.617–0.936)	7.000	0.643 (0.351–0.872)	0.846 (0.546–0.981)	0.818 (0.482–0.977)	0.687 (0.404–0.895)
Gait (med-off)	0.788 (0.589–0.921)	2.000	0.857 (0.572–0.982)	0.692 (0.386–0.909)	0.750 (0.476–0.927)	0.818 (0.482–0.977)
QSM (caudate)	0.885 (0.703–0.975)	0.037	0.714 (0.419–0.916)	1.000 (0.753–1.000)	1.000 (0.692–1.000)	0.765 (0.501–0.932)
QSM (SN)	0.709 (0.503–0.866)	0.073	0.857 (0.572–0.982)	0.615 (0.316–0.861)	0.706 (0.440–0.897)	0.800 (0.444–0.975)
Combination ^#^	0.901 (0.724–0.982)	0.743	0.714 (0.419–0.916)	1.000 (0.753–1.000)	1.000 (0.692–1.000)	0.765 (0.501–0.932)

Data are presented as the number or result (confidence interval). # indicates the prediction probability of a binary logistic regression model combining axial symptoms (med-off) and the average QSM value in the caudate for the PSDXb levels. PSDXb = the ratio of the power spectral density of beta oscillations to that of the LFP signals, AUC = area under curve, CI = confidence interval, NPV = negative prediction value, PPV = positive prediction value, QSM = quantitative susceptibility mapping, SN = substantia nigra.

## Data Availability

The data that support the findings of this study are available from the corresponding authors upon reasonable request.

## Supplementary Materials

The following supporting information can be downloaded at https://www.mdpi.com/article/10.3390/brainsci13010081/s1, Table S1: Clinical and demographic information of the PD patients and healthy controls; Table S2: Comparison of QSM values between PD patients and HC subjects; Table S3: Results of the correlation analysis between left and right PSDXb and QSM values in ipsilateral DGM structures; Table S4: Results of the correlation analysis between bilateral average QSM values and UPDRS part III; Figure S1: Motor cortex circuitry activity changes in Parkinson disease.
