# Supplementary material for: Association between Beta Oscillations from Subthalamic Nucleus and Quantitative Susceptibility Mapping in Deep Gray Matter Structures in Parkinson’s Disease
Source: Brain Sci. 2023 Jan 1;13(1):81. doi: 10.3390/brainsci13010081 (PMC9857066; doi:10.3390/brainsci13010081)
Supplement: Supplementary file 1 [file brainsci-13-00081-s001.zip › brainsci-2007159-supplementary.pdf]

Supplementary data

Table S1. Clinical and demographic information of the PD patients and healthy controls

Clinical features	PD patients (n=27)	HC subjects (n=27)	*p*
Age (year)	60.0 (55.3 ~ 64.0)	61.0 (55.5 ~ 63.5)	0.897
Gender (male/female)	16/11	12/15	0.587
Disease duration (years)	9.0 (7.0 ~ 12.0)	NA	NA
LEDD (mg)	798 (589 ~ 1036)	NA	NA
H-Y	3.0 (3.0 ~ 3.0)	NA	NA
Preoperative UPDRS part III			
Med-off	53.0 (45.3 ~ 59.8)	NA	NA
Med-on	25.0 (19.8 ~ 29.8)	NA	NA

Data were presented as number or median (interquartile range). HC = healthy control, H-Y = Hoehn and Yahr stages, MMSE = Mini-Mental State Examination, PD = Parkinson's disease, UPDRS = Unified Parkinson's Disease Rating Scale.

Table S2. Comparison of QSM values between PD patients and HC subjects

Bilateral average QSM value (ppm)	PD patients (n=27)	HC subjects (n=27)	Z	*p*
Caudate	0.035(0.031~0.038)	0.035(0.028~0.039)	-0.251	0.802
GPi	0.068(0.059~0.077)	0.067(0.064~0.075)	-0.147	0.883
GPe	0.091(0.078~0.101)	0.086(0.081~0.098)	-0.303	0.762
Putamen	0.038(0.034~0.043)	0.035(0.031~0.044)	-0.735	0.462
STN	0.063(0.055~0.075)	0.065(0.059~0.069)	-0.303	0.762
SN	0.080(0.070~0.100)	0.074(0.067~0.078)	-1.981	**0.048***
RN	0.074(0.068~0.081)	0.068(0.059~0.075)	-2.361	**0.018***
Dentate	0.057(0.048~0.066)	0.060(0.051~0.067)	-0.389	0.697

Data were presented as median (interquartile range). *, represented a statistical difference ($P<0.05$). QSM = quantitative susceptibility mapping, PD = Parkinson's disease, HC = healthy control, GPi = internal globus pallidus, GPe = external globus pallidus, STN = subthalamic nucleus, SN = substantia nigra, RN = red nucleus, DN = dentate nucleus.

Table S3. Results of the correlation analysis between left or right PSDXb and QSM values in ipsilateral DGM structures

QSM value	Ipsilateral PSDXb		
	rho	*p*	*p* (FDR corrected)
Left side			
Caudate	0.555	**0.003***	**0.024***
GPi	0.335	0.088	0.176
GPe	0.352	0.072	0.176
Putamen	0.220	0.269	0.359
STN	0.266	0.181	0.290
SN	0.399	0.039	0.156
RN	0.036	0.858	0.961
DN	0.010	0.961	0.961
Right side			
Caudate	0.546	**0.003***	**0.024***
GPi	0.300	0.128	0.205
GPe	0.350	0.073	0.146
Putamen	0.213	0.286	0.381
STN	0.411	0.033	0.088
SN	0.463	0.015	0.060
RN	0.010	0.959	0.959
DN	-0.189	0.346	0.395

* represented a statistical correlation ($p<0.05$). DGM = deep gray matter, PSDXb = the ratio of power spectral density of beta oscillations to that of the LFP signals, FDR = false discovery rate, QSM = quantitative susceptibility mapping, GPi = internal globus pallidus, GPe = external globus pallidus, STN = subthalamic nucleus, SN = substantia nigra, RN = red nucleus, DN = dentate nucleus.

Table S4. Results of the correlation analysis between bilateral average QSM values and UPDRS part III

Bilateral average QSM value	UPDRS part III (med-off)			UPDRS part III (med-on)		
	rho	P	*p* (FDR corrected)	rho	*p*	*p* (FDR corrected)
Caudate	-0.296	0.133	0.709	-0.296	0.133	0.709
GPi	-0.024	0.906	0.906	-0.024	0.906	0.906
GPe	-0.337	0.085	0.709	-0.337	0.085	0.709
Putamen	-0.141	0.483	0.906	-0.141	0.483	0.906
STN	-0.227	0.255	0.906	-0.227	0.255	0.906
SN	-0.154	0.443	0.906	-0.154	0.443	0.906
RN	0.034	0.868	0.906	0.034	0.868	0.906
DN	0.314	0.110	0.709	0.314	0.110	0.709

* represented a statistical correlation ($p<0.05$). QSM = quantitative susceptibility mapping, UPDRS = Unified Parkinson's Disease Rating Scale, FDR = false discovery rate, GPi = internal globus pallidus, GPe = external globus pallidus, STN = subthalamic nucleus, SN = substantia nigra, RN = red nucleus, DN = dentate nucleus.

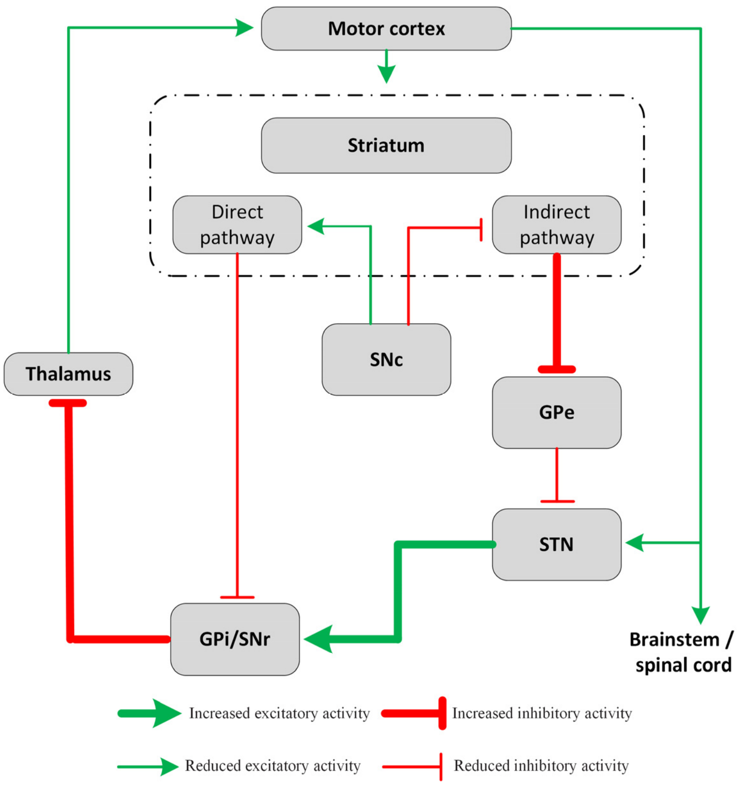

Figure S1. Motor cortex circuitry activity changes in Parkinson disease.

SNc = Substantia nigra pars compacta, GPe = external globus pallidus, GPi = internal globus pallidus, SNr = Substantia nigra pars reticulate, STN = Subthalamic nucleus.
